# Synergistic action of the gut microbiota in environmental RNA interference in a leaf beetle

**DOI:** 10.1186/s40168-021-01066-1

**Published:** 2021-05-04

**Authors:** Letian Xu, Shijing Xu, Liuwei Sun, Yiqiu Zhang, Jing Luo, Ralph Bock, Jiang Zhang

**Affiliations:** 1 State Key Laboratory of Biocatalysis and Enzyme Engineering, School of Life Sciences, Hubei University, Wuhan, 430062 China; 2 Max-Planck-Institut für Molekulare Pflanzenphysiologie, Am Mühlenberg 1, D-14476 Potsdam-Golm, Germany

**Keywords:** Gut bacteria, RNA interference, Double-stranded RNA, Leaf beetle, Synergism

## Abstract

**Background:**

RNA interference (RNAi) has emerged as an efficient tool to control insect pests. When insects ingest double-stranded RNAs (dsRNAs) targeted against essential genes, strong gene silencing and mortality can be induced. To exert their function, dsRNA molecules must pass through the insect’s gut and enter epithelial cells and/or the hemolymph. Gut bacteria are known to play multifarious roles in food digestion and nutrition, and confer protection against pathogens and parasites. Whether there is a cross talk between gut bacteria and ingested dsRNAs and whether the microbiome affects RNAi efficiency are unknown.

**Results:**

Here, using a leaf beetle gut microbiota system, we investigated whether gut bacteria interact with dsRNA molecules and how the gut microbiota affects RNAi responses in insects. We first showed that the leaf beetle *Plagiodera versicolora* (Coleoptera) is highly susceptible to RNAi. We then demonstrated that ingestion of dsRNAs by non-axenic *P. versicolora* larvae results in (i) significantly accelerated mortality compared with axenic larvae, and (ii) overgrowth and dysbiosis of the gut microbiota. The latter may be caused by bacterial utilization of dsRNA degradation products. Furthermore, we found that *Pseudomonas putida*, a gut bacterium of *P. versicolora*, acts as major accelerator of the death of *P. versicolora* larvae by transitioning from commensal to pathogenic lifestyle.

**Conclusions:**

The present study illuminates the complex interplay between lethal dsRNA, the insect host, and its gut microbiota. The ingestion of dsRNA by the leaf beetle caused a dysbiosis of gut bacterial community, and the dsRNA degradation products by host insect preferentially promoted the growth of an entomopathogenic bacterium, which accelerated dsRNA lethality to the insect. Our findings reveal a synergistic role of the gut microbiota in dsRNA-induced mortality of pest insects, and provide new insights in the mechanisms of RNAi-based pest control.

**Video abstract**

**Supplementary Information:**

The online version contains supplementary material available at 10.1186/s40168-021-01066-1.

## Background

RNA interference (RNAi) is a conserved post-transcriptional gene-silencing process in which gene expression in eukaryotic cells is downregulated by double-stranded RNAs (dsRNAs) and their degradation intermediates. RNAi has been widely used as a powerful experimental tool for determining gene functions, and more recently, was repurposed as a novel strategy for insect pest control. When dsRNAs that target essential insect genes are ingested by the pest insect, knockdown of the target genes by RNAi can result in reduced growth or even death [1]. RNAi-based pest control is, therefore, recognized as a promising approach to develop new target-specific pesticides. Coleopteran insects, such as the red flour beetle (*Tribolium castaneum*), the western corn rootworm (*Diabrotica virgifera virgifera*), and the Colorado potato beetle (*Leptinotarsa decemlineata*) are highly susceptible to RNAi and can be efficiently killed, if lethal dsRNAs are orally delivered. Interestingly, the efficiency of RNAi-mediated pest control was shown to be highly variable among different groups of insects [2–5]. A few factors influencing the RNAi efficiency in insects have been identified, including the stability of dsRNA in the digestive system, the cellular uptake efficiency of dsRNAs, endosomal entrapment, the activity of the core RNAi machinery, and geographic variation [6–9]. However, a comprehensive understanding of the molecular mechanisms affecting RNAi efficiency is still lacking. Moreover, the complex network of molecular interactions underpinning the dsRNA-mediated killing mechanisms has received limited attention.

The gut of insects provides a distinctive and complex environment for microbial colonization. The gut microbiota in some insects offers various beneficial services to the host, including multifarious contributions to food digestion, nutrient modification, pathogen defense, and chemical detoxification [10]. In addition to the positive contribution to the insect’s fitness, the presence of gut bacteria has also been implicated in promoting the insecticidal activity of *Bacillus thuringiensis* [11–14]*.* Furthermore, it was demonstrated that some commensal gut bacteria have the potential to translocate from the gastrointestinal tract into the hemolymph and induce rapid death [15–17]. Upon ingestion of dsRNA by insects, the dsRNA molecules inevitably pass through the intestinal tract, where they get in contact with the diverse microbial community that thrives in the gut. Whether there is an interplay between the gut microbiota and exogenously applied dsRNAs and whether or not the microbiome affects the RNAi response of insect are largely unknown.

The willow leaf beetle, *Plagiodera versicolora*, is one of the most damaging pest species of Salicaceae plants such as willows and poplars [18]. This pest is widely distributed across northern Africa, America, Europe, and Asia. *P. versicolora* larvae and adults both feed on leaves of willows and poplars, especially during the summer months. If not controlled, the insects can completely skeletonize the leaves and cause serious economic damage [19].

In the present work, we have studied the response of *P. versicolora* to environmental RNAi. We found that, like many other Coleopteran insects, *P. versicolora* is susceptible to RNAi, and can be efficiently killed by feeding with dsRNAs against essential genes. We further explored the role of the gut microbiota in the RNAi response in *P. versicolora*. Our results indicate that the gut microbiota can utilize the dsRNA degradation products for proliferation, and contribute substantially to insect mortality.

## Methods

### Preparation of *P. versicolora* larvae and gut bacterial strains

*P. versicolora* insects used in the studies were regularly reared by feeding with detached fresh willow leaves at 28 °C and 60 ± 5% relative humidity under a 16-h light /8-h dark photoperiod. Newly laid eggs or hatched larvae were removed from the willow leaves and transferred to axenic poplar leaves to conduct the experiments (at 28 °C, 60% ± 5% relative humidity, 16 h daylength). Poplar plantlets were raised aseptically on a synthetic medium containing 4.4 g/L Murashige & Skoog (MS) salts plus vitamins, 0.1 mg/L NAA and 3% (w/v) sucrose in Magenta boxes [20]. Aseptic poplar plantlets were originally produced by surface sterilization of a stem of a poplar plant grown in the greenhouse. Endophytes were removed by tissue dissection and culture of shoot apical meristems (SAM) in the same MS-based medium. To obtain axenic larvae, egg masses were soaked in 40% NaOH for 1 min and subsequently in 70% ethanol for 5 min, and finally washed with sterilized water for 2 min. After air-drying, each egg was separately transferred onto Luria-Bertani (LB) solid medium. Removal of gut bacteria was confirmed by a colony-forming unit assay and PCR analysis using conserved primers for the 16S rRNA gene of gut bacteria. The newly hatched larvae were fed with aseptic detached poplar leaves and maintained axenically in Petri dishes sealed with Parafilm. To evaluate the influence of the removal of gut microbiota on *P. versicolora*, 30 axenic and non-axenic larvae were fed with aseptic poplar leaves that were replaced every day, and the survival rate was recorded daily. Another group of identically treated larvae was used for body mass determination and weighed daily.

Strains of the gut bacteria *Enterobacter aerogenes* (accession number: MT835160)*, Pseudomonas putida* (accession number: MT791338), and *Enterococcus faecalis* (accession number: MT791337) were isolated from *P. versicolora*. For isolation of gut bacteria, individual guts of *P. versicolora* larvae (*n* = 15, second instar) were dissected and crushed in 100 μL of 10% PBS. After sonication for 1 min, the suspension was vortexed for 10 s, and the diluted suspension (10^2^ to 10^4^) was plated on LB solid medium, followed by incubation at 28 °C for 12–48 h [21]. Morphologically different colonies were selected and streaked for purification. To identify the bacterial species, samples of genomic DNA from the different bacterial isolates were obtained by extraction with the kit Roche, USA and used to amplify 16S rRNA gene sequences by PCR using the universal 16S rRNA gene-specific primers 27F and 1492R (Table S1). The purified PCR products were then sequenced (Sangon Biotech, Shanghai, China), and the obtained sequences were aligned with the closest relatives matching the 16S rRNA gene sequences by BLAST searches (*http://blast.ncbi.nlm.nih.gov/Blast.cgi*).

### Ingestion and injection of dsRNA in *P. versicolora* larvae

*Srp54k*, *Actin*, *Snap*, *Shi*, and *EGFP* gene fragments were amplified by PCR using gene-specific primer pairs (Table S1), and subsequently used as templates to synthesize dsRNA (ds*Srp54k*, ds*Actin*, ds*Snap*, ds*Shi*, and ds*GFP*) with the T7 RiboMAX^TM^ Express RNAi system (Promega, USA) following the manufacturer’s instructions. Integrity of dsRNAs was evaluated by electrophoresis in 1.5% agarose gels, and the amounts of dsRNA were quantified with a spectrophotometer (Nano-Drop 2000, Thermo Scientific, USA).

For dsRNA feeding assays, 30 second instar larvae were fed with aseptic poplar leaves that had been painted with 8 ng/cm^2^ dsRNA. The leaves were exchanged daily (by fresh poplar leaves coated with 8 ng/cm^2^ dsRNA), and survival was recorded. To examine the efficiency of RNAi-mediated knockdown of target genes, an additional group of (identically fed) larvae was raised; five larvae were randomly chosen each day after dsRNA feeding, and total RNA was isolated for further analysis. For axenic larvae, bacterial 16S rRNA gene expression was quantified by qRT-PCR to confirm their sterility.

For dsRNA injection assays, 30 second instar larvae were randomly chosen, and samples of 36 ng ds*Srp54k* or 2 ng ds*Actin* in 5 nL were injected into each larva using a micro-injector (World Precision Instruments, Sarasota, USA). Negative controls received an equivalent amount of ds*GFP* (36 ng ds*GFP* in the control group of ds*Srp54k* and 2 ng ds*Actin* in the control group of ds*Actin*, respectively). After injection, *P. versicolora* larvae were reared on sterile poplar leaves, and survival rates were monitored daily (*n* = 30). The experiment was repeated, and five larvae were randomly chosen each day for RNA extraction.

To suppress dsRNase activity, larvae were fed with fresh poplar leaves painted with 40 ng/cm^2^ ds*dsRNase* and 8 ng/cm^2^ ds*GFP* or, as a control, the equivalent amount of ds*GFP* (40 ng/cm^2^) and 8 ng/cm^2^ ds*GFP*. Five larvae were randomly chosen after 4 days of feeding for RNA extraction. To compare gene expression level of *dsRNase1* and *dsRNase2 in vivo*, standard curves were established from serial dilutions (10, 50, 250, and 1250 plasmid copies per microliter). Plasmid numbers were calculated based on the molecular weight of the plasmid, and the DNA concentration of the plasmid sample. The standard curves for *dsRNase1* and *dsRNase2* were *y* = − 3.549*x* + 35.953 (*R*^2^ = 0.999) and *y* = − 3.144*x* + 32.18 (*R*^2^ = 0.999), respectively. The efficiency of primers (*E*) was calculated using the equation *E* = 10 ^(-1/slope)^(*E*_*dsRNase1*_ is 91.3%, *E*_*dsRNase2*_ is 107.9%). Transcript numbers of the two genes in 1 ng cDNA were calculated based on the Cq value and the formula *n* = *E*^(intercept-Cq)^(*n* = 3) [22, 23].

### Analysis of gut microbiota

To mimic the situation in the natural environment, poplar leaves were harvested from plants of the same developmental stage and coated with 8 ng/cm^2^ of ds*Srp54K*, ds*Actin*, ds*GFP*, or H_2_O as a control. Second instar larvae were fed on these leaves, and the coated leaves were exchanged daily. After 3 days of feeding, larvae were disinfected in 70% ethanol for 10 s, rinsed in sterile water several times, and then dissected under sterile conditions. Four guts were pooled together as a replicate (*n* = 5). DNA was extracted from gut samples with the High pure PCR template preparation kit (Roche, USA). A 16S rRNA gene fragment encompassing the V3 and V4 hypervariable regions was amplified by PCR using the primer pair 338F and 806R (Table S1) in a reaction volume of 20 μL containing 10 ng of DNA, 5 μM of each primer, 2.5 mM dNTPs, 0.4 μL Fastpfu polymerase (Transgene, China), and 5 × Fastpfu buffer. Amplification reactions were performed in an ABI GeneAmp 9700 thermal cycler with an initial denaturation step at 95 °C for 2 min, followed by 27 cycles of annealing and extension (95 °C for 30 s followed by 55 °C for 30 s and an extension step at 72 °C for 30 s) and a final extension at 72 °C for 10 min. A DNA extraction blank and a PCR blank control were included, and yielded no product band on a denaturing gradient gel after 30 cycles of PCR amplification. Thus, the PCR blank control was not subjected to pyrosequencing.

Sequencing of 16S rRNA amplicons was performed using an Illumina MiSeq platform (Illumina, San Diego, USA) in MAJORBIO. Raw 16S rRNA gene sequencing reads were demultiplexed, quality filtered by Trimmomatic, and paired-end reads were assembled using FLASH (V1.2.7) with the following criteria: (1) the 300 bp reads were truncated at any site receiving an average quality score of < 20 over a 50 bp sliding window, and truncated reads shorter than 50 bp were discarded; (2) exact barcode matching, two nucleotide mismatches in primer matching, and reads containing ambiguous characters were removed; and (3) a minimum overlap of 10 base pairs (bp). High-quality data (clean reads) were analyzed using the QIIME 2 software package (Version 2020.6, *https://qiime2.org/*). The DADA2 pipeline was employed to identify actual sequence variants (ASVs), which produced an ASV table containing ~ 710,000 high-quality reads belonging to 211 ASVs. Sequences were rarefied to 24,393 reads per sample. Taxonomic assignment of ASVs was performed using the VSEARCH consensus taxonomy classifier in QIIME 2 and reference data sets from the SILVA 16S rRNA database. Microbial diversity and community composition were analyzed using vegan packages in R (version 3.5.1) [24]. Non-metric multidimensional scaling (NMDS) using the Jaccard similarity matrix and PCoA (principal coordinate analysis) based on Bray-Curtis dissimilarities were used to identify differences between microbial communities. Compositional differences in NMDS were tested using ANOSIM with 1000 permutations. A Permutational Multivariate Analysis of Variance based on the weighted UniFrac distance (PERMANOVA) was used to test for differences in PCoA between treatments. The raw pyrosequencing reads were obtained and deposited in the NCBI Sequence Read Archive under accession number SAMN15293221 (reference: BioProject PRJNA639897).

### *In vitro* incubation of dsRNA with hemolymph or gut juice

Since the second instar larva is too small for hemolymph and gut juice extraction, third instar larvae were used for these experiments. Hemolymph fluid samples were collected with a 10-μL glass capillary tube from abdomens of 10 third instar larvae (0.5–1.0 μL per larva) and were subsequently diluted with 100 μL ice-cold Ringer solution (1 L: 8.766 g NaCl; 0.188 g CaCl_2_; 0.746 g KCl; 0.407 g MgCl_2_; 0.336 g NaHCO_3_; 30.807 g sucrose; 1.892 g trehalose; pH 7.2) [25]. Samples were then centrifuged at 12,000 *g* for 10 min to remove hemocytes, and the supernatant was collected. For gut juice preparation, whole guts from 10 third instar larvae were dissected and then crushed in 100 μL ice-cold Ringer solution in a centrifuge tube, followed by centrifugation at 12,000 *g* for 10 min and collection of the supernatants. The total protein concentrations of the fluid samples were determined using the BCA Protein Assay kit following the manufacturer’s instructions. The amount of hemolymph and gut juice was normalized and adjusted to 1 μg/μL of total protein for the two samples. One hundred fifty nanograms of ds*GFP* (dissolved in 5 μL nuclease-free water) were added to 5 μL hemolymph or gut juice and incubated at 30 °C for the indicated times. Incubation in Ringer solution with the same amount of ds*GFP* served as control. Subsequently, 2 μL of 6× loading dye (20% glycerol, 1.25 mM Na_2_EDTA, 0.1% bromophenol blue, 0.1% xylene cyanol) was applied to the 10-μL samples, and the RNAs were analyzed by electrophoreses in 1% agarose gels containing Gelview.

### Bacterial growth promotion and inhibition assays

To measure the effects of dsRNA degradation products on growth of gut bacteria, bacterial cultures were grown in a modified minimal medium [26]. For carbon source determination, bacteria were grown in minimal medium supplemented with 24 nmol/mL ds*GFP*, ribose, adenine, adenosine, cytidine, uridine, or inosine as sole carbon source and 0.4% (NH_4_)_2_SO_4_ as nitrogen source. Guanine and uracil are not sufficiently soluble, and, therefore, were not included in the tests. For nitrogen source determination, bacteria were grown in minimal medium supplemented with 4% glucose and 0.05% sodium citrate as carbon source and 24 nmol/mL ds*GFP*, ribose, adenine, adenosine, cytidine, uridine, or inosine as sole nitrogen source. Saline-washed cells (approximately 10^7^ cells) of each bacterial strain were used for inoculation. Subsequently, the cultures were shaken for 24 h at 200 rpm and 30 °C. The bacterial cell number was determined by counting colony-forming units (CFUs) on LB medium.

### Reintroduction of bacteria into insect guts

After overnight culture, bacteria (*E. aerogenes*, *P. putida*, and *E. faecalis*) were collected by centrifugation at 4000 rpm for 5 min to remove the supernatant. The bacterial cells were then washed with sterile PBS 3 times, resuspended in PBS, and diluted to a final concentration of approximately 1 × 10^6^ cells/mL [27]. The bacterial suspension was mixed with the indicated amounts of dsRNA and painted onto aseptic poplar leaves. The leaves were then fed to second instar axenic *P. versicolora* larvae (*n* = 40). The aseptic poplar leaves coated with bacteria (approximately 1 × 10^4^ cells/cm^2^) and dsRNA (8 ng/cm^2^) were replaced every day. Survival was recorded daily.

### Quantitative real-time PCR analysis

RNA was extracted using RNAiso plus as described by the manufacturer (Takara, Japan). cDNA was synthesized from 2 μg of total RNA using the Hifair^@^ 1^st^ Strand cDNA Synthesis for qPCR Kit (YEASEN, China) with random primers. qPCRs were performed in an Applied Biosystems 7300 Real-Time PCR system using SYBR premix Ex Taq^TM^ (Takara, Japan). cDNA templates were denatured at 95 °C for 2 min, followed by 40 two-segment cycles of amplification at 95 °C (5 s) and 60 °C (30 s), where the fluorescence was automatically measured. A melting curve analysis was performed after the qPCR run (between 65 °C and 95 °C with 0.5 °C increments). Prior to use in qRT-PCR, cDNA was 1:9 diluted with H_2_O. All data were normalized to levels of a housekeeping gene (5S rRNA gene), and gene expression levels were calculated as fold change values using the 2^-ΔΔCt^ method, except for the comparison of *dsRNase1* and *dsRNase2* [28]. Each experiment was carried out in triplicate. The primers used for the quantification of the three genera (*Enterobacter*, *Pseudomonas*, *Enterococcus*) are based on sequences used in previous studies [29–31]. Other primers used were designed by Primer Premier 6. Primer sequences are listed in Table S1.

### Histological analysis

The whole larval body was immediately fixed in 10% neutral buffered formalin supplemented with 2% dimethyl sulfoxide for 24 h, then dehydrated in a series of alcohol baths (beginning with 50% and progressing to 100%), cleared in xylol for 4 h, and finally embedded in paraffin. Cross sections were prepared with a microtome (LEICA RM 2016, Leica Microsystems, USA) at a thickness of about 3 μm, stained with hematoxylin and eosin, and analyzed with a fluorescence microscope (Nikon Eclipse E-200 model, Tokyo, Japan).

### Statistical analysis of data

Prior to analysis, all variables were tested for normality with the Kolmogorov–Smirnov test. Data comprising two groups were analyzed using Student’s *t* test for unpaired comparisons, and data comprising more than two groups were analyzed using one-way ANOVA coupled with Bonferroni (equal variances) or Dunnett’s T3 (unequal variances) correction multiple comparison test. Survival curves were analyzed using the Kaplan–Meier method, and the log-rank test was used to evaluate the significance of differences between two groups. A value of *P* < 0.05 was considered significantly different. Data were statistically analyzed using SPSS version 19.0. Figures were drawn using Origin 8.5 and GraphPad Prism 7, and assembled in Adobe Illustrator CS6 and Photoshop CS6.

## Results

### Gut microbiota accelerate dsRNA-induced mortality of *P. versicolora* larvae

We first wanted to examine the sensitivity of *P. versicolora* to RNAi and identify essential genes that represent suitable targets for RNAi-mediated pest control [32, 33]. To this end, larvae were fed with *in vitro* synthesized dsRNAs targeted against the *β-Actin*, *Srp54k*, *Snap*, and *Shi* genes. *β-Actin* encodes a multi-functional protein for microfilament formation [34]. *Srp54k* encodes the 54 kDa subunit of the signal recognition particle which is involved in protein targeting to the endoplasmic reticulum [35]. *Snap* encodes the alpha-soluble N-ethylmaleimide-sensitive factor attachment protein that is involved in the docking and fusion of vesicles to target membranes [36]. *Shi* encodes a dynamin-like protein associated with vesicular trafficking [37]. Compared with the negative control (feeding with *GFP* gene-derived dsRNA; ds*GFP*), feeding of *P. versicolora* with dsRNAs derived from *Srp54k, Actin*, and *Snap* resulted in 100% mortality of the larvae. The effects of RNAi were shown to be ds*Actin* > ds*Srp54k* > ds*Snap* (Figure S1; log-rank test, *P <* 0.05).

To evaluate whether the gut microbiota of *P. versicolora* is involved in determining the efficiency of dsRNA-mediated killing, axenic larvae were obtained by surface sterilization of eggs. The hatched larvae were then reared on aseptically grown poplar leaves. The axenic status of the larvae was confirmed by two independent methods: (i) absence of bacterial colony formation on LB agar plates and (ii) absence of PCR products from amplification reactions using primers (primer pair 338F and 806R; Table S1) for conserved regions of the bacterial 16S rDNA (Figure S2a,b). The axenic growth had no influence on insect survival and body mass compared with conventionally raised larvae (Figure S2c, d).

**Fig. 1:**
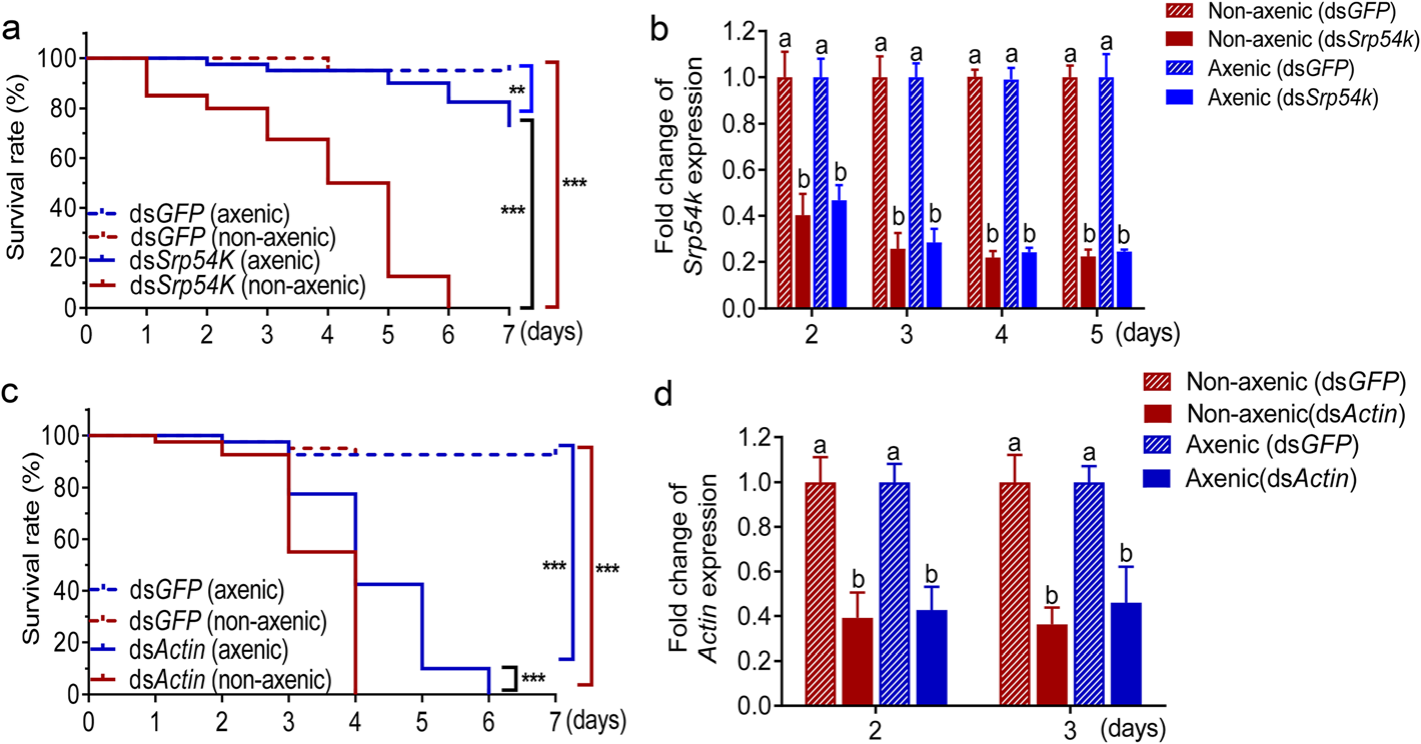
Gut bacteria accelerate mortality of *P. versicolora* in dsRNA-mediated environmental RNAi. **a** and **c** Kaplan–Meier survival curves of axenic and non-axenic larvae after feeding with ds*Srp54k* (**a**) and ds*Actin* (**c**) (*n* = 30/group, shown is one representative experiment of four repeats). *Srp54k* (**b**) and *Actin* (**d**) relative expression in axenic and non-axenic larvae fed with ds*Srp54k* and ds*Actin* (*n* = 5). Gene expression levels were set as one in larvae fed with ds*GFP* control. Kaplan–Meier survival curve was analyzed by the log-rank test. Gene expression differences between two groups were calculated using the independent samples *t* test. Data are presented as mean ± SE, ^***^ *P* < 0.001; ^**^ *P* < 0.01; different letters indicate significant difference (*P* < 0.05)

dsRNA feeding assays revealed no significant differences in survival of axenic larvae compared with non-axenic larvae when fed with ds*GFP*. By contrast, non-axenic larvae fed with ds*Srp54k* or ds*Actin* were killed significantly faster than axenic larvae (Fig. 1a,c; log-rank test, *P* < 0.001). The axenic status of the larvae was confirmed by qPCR (see Methods section). Surprisingly, no significant differences in the level of gene silencing were found between axenic and non-axenic larvae (Fig. 1b,d, *P* < 0.05). This finding suggests that the striking differences in mortality between non-axenic and axenic larvae fed with lethal dsRNAs are not caused by differences in the strength of the suppression of the targeted genes. Instead, these results raise the interesting possibility that the gut microbiota are involved in the acceleration of dsRNA-mediated mortality in non-axenic *P. versicolora* larvae.

To examine whether breakdown of the barrier function of the gut epithelium is responsible for the enhanced killing efficiency, we isolated hemolymph from non-axenic larvae fed with dsRNAs and assayed the hemolymph samples for the presence of bacteria by culturing them on LB medium. While numerous bacterial colonies formed from non-axenic larvae fed with ds*Srp54k* or ds*Actin*, no colonies grew from hemolymph samples obtained from larvae fed with ds*GFP*. This finding indicates translocation of gut bacteria to the hemocoel in *P. versicolora* larvae fed with lethal dsRNAs (ds*Srp54k* or ds*Actin*; Figure S3). Moreover, histological analysis revealed that the gut epithelia of non-axenic larvae fed with lethal dsRNAs were disrupted, while they remained intact in the ds*GFP* control (Figure S4). These observations provide further evidence for gut bacteria promoting dsRNA-mediated killing.

### Feeding of dsRNAs results in alteration of the gut microbiota in *P. versicolora*

We next investigated the composition of the gut bacterial community in *P. versicolora* and its change after ingestion of lethal dsRNAs (ds*Srp54k* or ds*Actin*) or ds*GFP* as control. Ingestion of sterilized water served as negative control. Amplified bacterial 16S rRNA sequences were determined by next-generation sequencing (see Methods section). Average read counts per sample between 355 and 677 were obtained, and the sequences were grouped into 211 ASVs. Alpha diversity was estimated using four measurements: ACE index, Chao1 index, Shannon–Weiner index, and Simpson’s index (Table S2). In general, no significant differences were found for the α-diversity indices between gut samples of control animals and the three groups of larvae feeding on dsRNA-painted leaves. However, the larvae fed with ds*Actin* had a significantly greater value for the Shannon diversity index and Simpson’s index than the other treatments (Table S2; one-way ANOVA, *P* < 0.05), possibly suggesting that the oral administration of ds*Actin* can decrease bacterial community evenness and increase species richness.

**Fig. 2:**
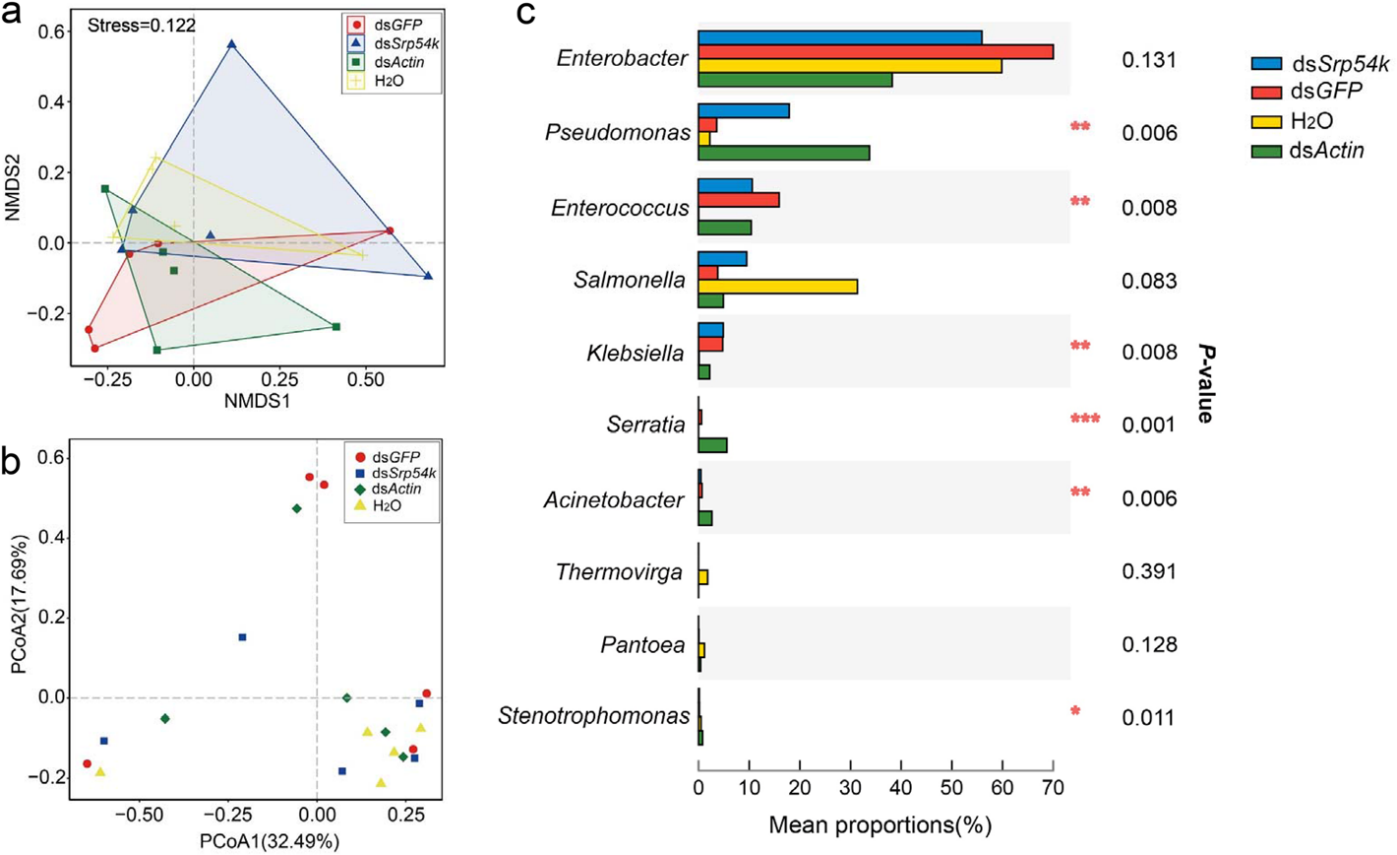
Ingestion of dsRNA alters gut microbiota composition of *P. versicolora*. **a** Non-metric multidimensional scaling (NMDS) diagrams of 20 samples, based on Jaccard matrix. **b** Principal coordinates analysis (PCoA) with Bray–Curtis dissimilarity of the bacterial community. **c** Kruskal–Wallis *H* test bar plot analysis of the relative proportions of major genera of gut bacteria (relative abundances of the top 10 genera). The relative abundances of the identified microbial taxa were determined in gut samples collected from larvae fed with ds*GFP*, ds*Srp54k*, ds*Actin*, or H_2_O. *P* values were calculated using the Kruskal–Wallis *H* test. ^***^ *P* < 0.001; ^**^ *P* < 0.01; ^*^ *P* < 0.05

NMDS ordination based on Jaccard distances showed that bacterial communities of the four treatments clustered together (ANONISM, *P* > 0.05). Principal coordinates analysis (PCoA) of Bray–Curtis distances of microbial communities showed a similar pattern (Fig. 2a; PERMANOVA, *P* > 0.05). In all samples, the gut bacterial community was dominated by four bacterial genera: *Enterobacter*, *Pseudomonas*, *Enterococcus*, and *Salmonella*, which account for over 95% of the total sequences in the samples (Fig. 2c; Table S3). The proportion of *Pseudomonas* and *Enterococcus* was significantly increased in the three groups of dsRNA-fed larvae compared with the control group (larvae feeding on dsRNA-free leaves) (Fig. 2c; Kruskal–Wallis *H* test, *P* < 0.05), and the proportion of *Enterobacter* decreased after feeding with the dsRNAs. Remarkably, feeding with lethal dsRNAs (i.e., ds*Srp54k* or ds*Actin*) significantly increased the proportion of *Pseudomonas* in *P. versicolora* larvae compared with larvae fed with untreated or ds*GFP*-treated leaves (Fig. 2c; Kruskal–Wallis *H* test, *P* < 0.05).

### Ingestion of dsRNA promotes growth of gut bacteria

**Fig. 3:**
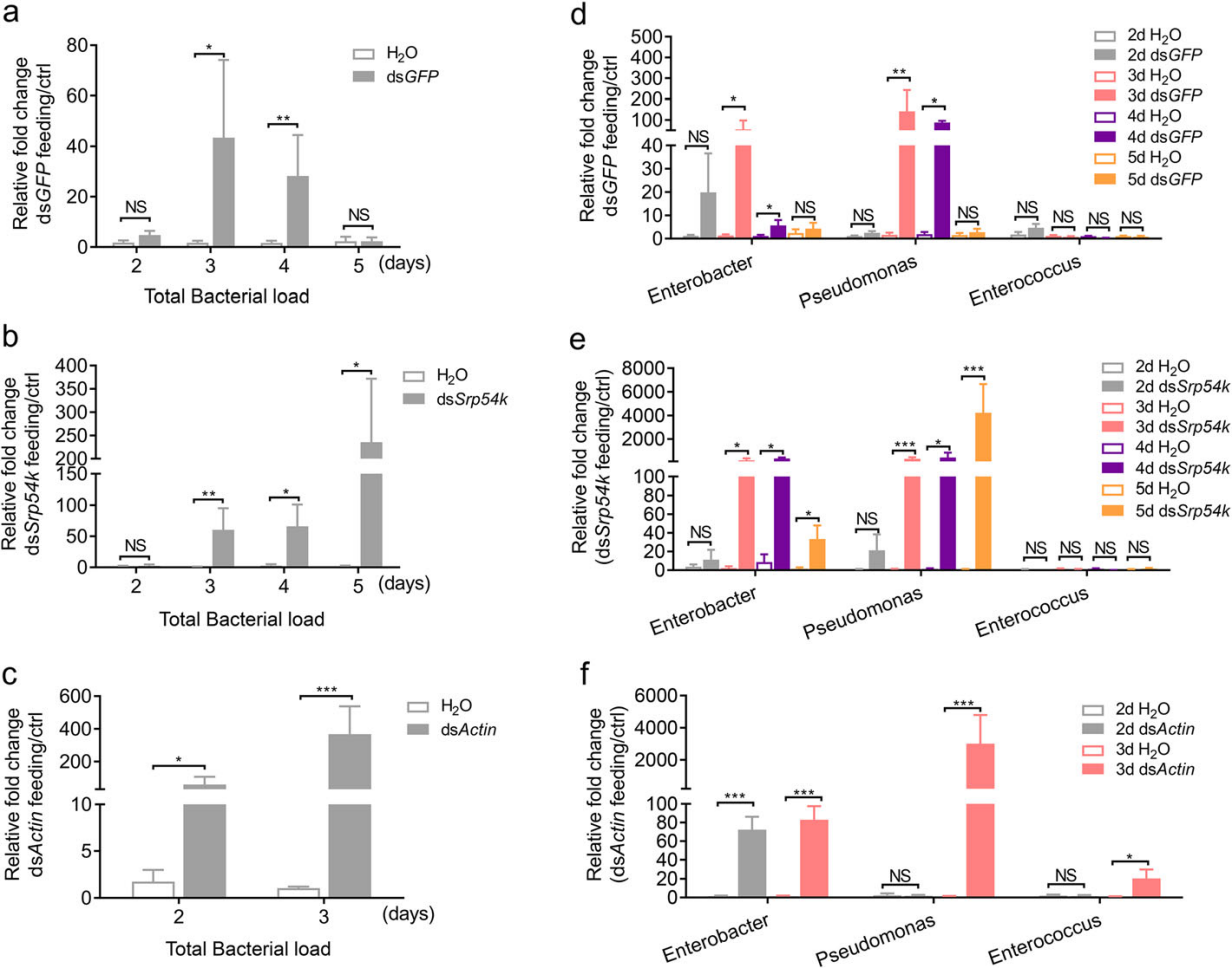
Ingestion of dsRNA promotes the growth of gut bacteria in *P. versicolora* larvae. qRT-PCR analyses were performed to determine the relative abundance of gut bacteria in *P. versicolora* larvae fed with ds*GFP* (**a**), ds*Srp54k* (**b**), and ds*Actin* (**c**) (*n* = 5), and the abundances of three major bacterial genera in non-axenic *P. versicolora* larvae fed with ds*GFP* (**d**), ds*Srp54k* (**e**), and ds*Actin* (**f**). The measurements were performed at different time points (after 2–5 days of feeding). The qRT-PCR value obtained for gut bacterial 16S rRNA in *P. versicolora* larvae fed with water-treated leaves was set as control. *P* values were calculated using the independent samples *t* test. Data are presented as means ± SE, ^***^ *P* < 0.001; ^**^ *P* < 0.01; ^*^ *P* < 0.05; *NS* not significant

The total amounts of gut bacteria were significantly increased in all dsRNA-fed larvae (2 days after feeding with ds*GFP* and ds*Actin*, 3 days after feeding with ds*Srp54k*) compared with the control group of larvae feeding on leaves not painted with dsRNA (Fig. 3a–c; *t* test, *P* < 0.05). This finding suggests that ingestion of dsRNA can promote bacterial growth in the intestinal system. To further validate the effects of dsRNA ingestion on the abundance of the three major genera of gut bacteria, we monitored the relative quantities of the bacterial genera in the different groups of larvae. The abundances of *Enterobacter* and *Pseudomonas* were found to be significantly increased in larvae fed with ds*GFP*-treated leaves at days 3 and 4 compared with the control (Fig. 3d; *t* test, *P* < 0.05), but dropped to similar abundance at day 5. The relative abundances of both *Pseudomonas* and *Enterobacter* were significantly increased after 3 days of feeding on ds*Srp54k*, while no significant differences were found in relative abundance of *Enterococcus* between the larvae fed with ds*Srp54k* and the control (Fig. 3e; *t* test, *P* < 0.05). Similarly, the relative abundances of both *Pseudomonas* and *Enterococcus* were significantly increased after 3 days of ds*Actin* feeding (Fig. 3f; *t* test, *P* < 0.05).

### Ingestion of dsRNA confers accelerated mortality and increased gut bacterial load in *P. versicolora*

**Fig. 4:**
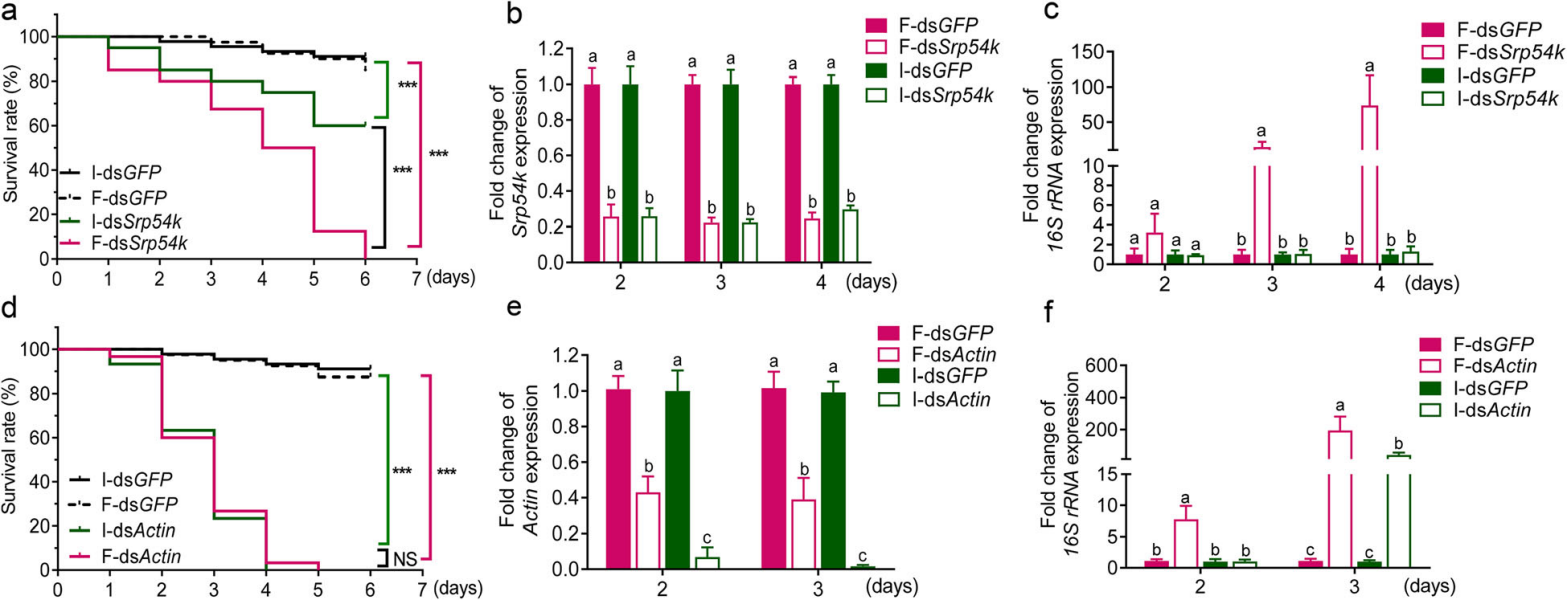
Comparison of mortality of *P. versicolora* in response to lethal dsRNA upon administration by injection (injected dsRNA, I-dsRNA) or ingestion (fed dsRNA, F-dsRNA). **a**, **d** Kaplan–Meier survival curves of *P. versicolora* larvae (*n* = 30) after injection and ingestion of ds*Srp54k* (**a**) or ds*Actin* (**d**). Ingestion or injection of ds*GFP* were performed as controls. The survival curves were analyzed by the log-rank test. **b**, **e** Expression levels of *Srp54k* and *Actin* in *P. versicolora* larvae fed or injected with ds*Srp54k* (**b**) or ds*Actin* (**e**). **c**, **f** qRT-PCR analysis of the relative abundance of whole gut bacteria in *P. versicolora* larvae after injection with ds*Srp54k* (**c**) or ds*Actin* (**f**) compared with ds*GFP* as control. *P* values were calculated using the independent samples *t* test. ^***^ *P* < 0.001; *NS*, not significant; Different letters indicate significant difference (*P* < 0.05)

To assess the contribution of ingestion of lethal dsRNA to the proliferation of gut bacteria, we determined mortality and bacterial loads of *P. versicolora* larvae after feeding and injection of dsRNAs. Upon injection of dsRNA, a significantly accelerated mortality was observed in larvae treated with ds*Srp54k* compared with those injected with ds*GFP* (Fig. 4a; log-rank test, *P <* 0.05). Moreover, we found that non-axenic larvae fed with ds*Srp54k* exhibit higher mortality and gut bacterial loads than larvae injected with ds*Srp54k* (Fig. 4a; log-rank test, *P <* 0.05; Fig. 4c, *t* test, *P <* 0.05), although the silencing levels of the target gene (*Srp54k*) were similar in the two treatments (Fig. 4b). Ingestion of ds*Actin* by *P. versicolora* larvae led to a similar mortality as injection of ds*Actin* (Fig. 4d), even though the latter treatment caused a significantly higher efficiency of gene silencing (Fig. 4e; *t* test, *P <* 0.05) and a lower gut bacterial load (Fig. 4f, *t* test, *P <* 0.05). Taken together, these results indicate that ingestion of ds*Srp54k* accelerates mortality of *P. versicolora* larvae, but the effect is not found in ds*Actin* feeding larvae. Similar mortality between ingestion and injection of ds*Actin* may be due to less efficient RNAi that could be counterbalanced by the role of commensal bacteria in causing lethality.

In addition to quantification of the bacterial amount, we monitored the immune responses of the different treatments by quantifying the expression of marker genes (*Duox*, *Attacin*) of immunity (Figure S6). These analyses revealed that larvae fed with ds*GFP* and ds*Srp54k* can sustain higher levels of immune gene expression than larvae fed with ds*Actin*. High-level immune gene expression may be responsible for the suppression of the relatively small increase in the bacterial load upon ds*GFP* feeding compared with ds*Actin* feeding, which is, however, not the case for ds*Srp54k* group. And the silencing of essential genes such as *Srp54k* and *Actin* likely severely impairs many physiological processes in the insect (Figure S4), which quickly leads to illness of the larvae, thus further limiting their capability to constrain gut bacterial overgrowth.

### dsRNA instability in *P. versicolora* gut juice

**Fig. 5:**
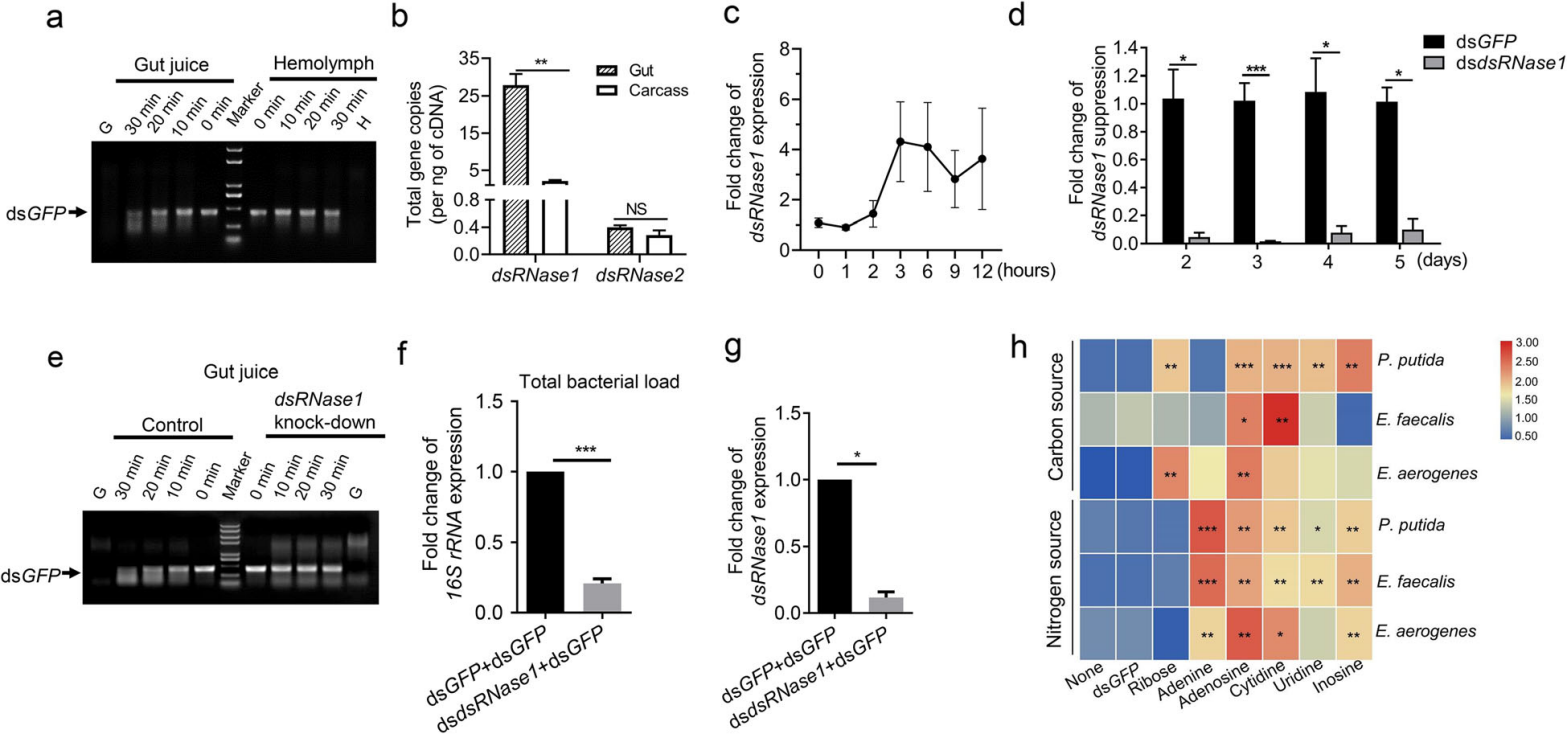
Degradation of dsRNA promotes gut bacterial growth in *P. versicolora* larvae. **a** ds*GFP* incubation with hemolymph or gut fluid of *P. versicolora* third instar larvae. Samples of 5 μL gut juice or hemolymph (adjusted to 5 μg of total protein) were incubated with 400 ng of ds*GFP* for the indicated times at 28 °C. G: gut juice sample without dsRNA added; H: hemolymph sample without dsRNA added. **b** Expression patterns of two putative *dsRNase* genes in third instar larvae. Each sample comprises five pooled guts or carcasses from dissected larvae. Error bars represent the mean of three independent samples ± SEM. **c** Relative expression changes of *dsRNase1* in larvae fed with ds*GFP*-painted leaves (8 ng/cm^2^) at the indicated times compared with untreated control larvae. Gene expression levels were set to 1 in larvae fed with H_2_O-treated control leaves (ctrl). **d** ds*RNase1* silencing efficiency over time in larvae (*n* = 5) injected with 40 ng of ds*dsRNase*. Injection of larvae with the same amount of ds*GFP* served as control. **e** ds*GFP* incubation in gut fluid of *P. versicolora* larvae with or without suppression of *dsRNase1* expression*.* Samples of 5 μL gut fluid (adjusted to 5 μg of total protein) were incubated with 400 ng of ds*GFP* for the indicated times at 28 °C. **f** qRT-PCR analysis of the relative abundance of gut bacteria in *P. versicolora* second instar larvae fed on young poplar leaves painted with ds*dsRNase1* (40 ng/cm^2^) + ds*GFP* (8 ng/cm^2^), or ds*GFP* (40 ng/cm^2^) + ds*GFP* (8 ng/cm^2^). Guts were sampled at day 4 (*n* = 7). **g** Relative expression levels of *d*s*RNase1* in the larvae assayed in (f). **h** dsRNA degradation products promote the growth of three gut bacterial species. Bacteria were grown for 24 h in either carbon-free or nitrogen-free liquid medium supplemented with 24 nmol/mL of the indicated compounds (dsGFP, ribose, adenine, adenosine, cytidine, uridine, inosine). Glucose (0.4%) and sodium citrate (0.05%) served as carbon source in nitrogen-free media. (NH_4_)_2_SO_4_ (0.4%) served as nitrogen source in carbon-free media. ^***^ *P* < 0.001, ^**^ *P* < 0.01, ^*^ *P* < 0.05

To obtain information on the stability of dsRNA in the *P. versicolora* digestive system and the hemolymph, ds*GFP* was incubated in gut juice or hemolymph extracted from insects. ds*GFP* was degraded faster in the gut juice than in the hemolymph (Fig. 5a). To determine the possible cause of dsRNA instability in the digestive tract, we retrieved two putative dsRNase sequences from our *P. versicolora* RNA-seq datasets. Both sequences have high sequence similarities to previously identified gut dsRNases (Figure S6). *dsRNase1* was found to be highly expressed in the gut tissue compared with the carcass, while expression of *dsRNase2* was relatively low in both gut and carcass, with no statistically significant difference between gut and carcass (Fig. 5b). Expression of *dsRNase1* is induced upon feeding of *P. versicolora* larvae with ds*GFP* (Fig. 5c). Importantly, the degradative capacity towards dsRNA of the gut juice of *P. versicolora* was reduced when *dsRNase1* was downregulated by injection of double-stranded RNA (ds*dsRNase1*) (Fig. 5d, e). These results indicate that dsRNase1 does a major dsRNA-degrading enzyme activity in the gut juice of *P. versicolora*.

### The degradation products of dsRNA can be utilized by gut bacteria for growth

To explore whether the instability of dsRNA contributes to the increased gut bacterial load, we fed larvae with dsRNA targeted against *dsRNase1* (ds*dsRNase1*) and dsGFP. For comparison, dsGFP was fed as a control. Interestingly, the gut bacterial load was significanly lower in the *dsRNase1*-silenced larvae than in larvae fed only with ds*GFP* (Fig. 5f, g), suggesting that dsRNA degradation is associated with increased gut bacterial load.

To futher assess whether dsRNA or its degradation products promote gut bacterial growth, we isolated and identified three bacterial species (*Enterococcus aerogenes, P. putida,* and *E. faecalis*) from the gut of *P. versicolora*, and used ds*GFP* and RNA degradation products (ribose, adenine, adenosine, cytidine, uridine, inosine) as either the sole carbon source or the sole nitrogen source for the growth of these bacterial species. As expected, ds*GFP* does not promote growth of any of the tested bacterial species. By contrast, all three species can utilize adenine, adenosine, cytidine, uridine, or inosine as sole nitrogen source. The growth of *P. putida* and *E. aerogenes* (but not *E. faecalis*) also can be stimulated with ribose as sole carbon source (Fig. 5h; Table S5). Only *P. putida* can use uridine and inosine as sole carbon source, although the bacterial biomass of *E. aerogenes* was also slightly increased when these compounds were fed as the sole carbon source (Fig. 5h).

### Reintroduction of gut bacteria into axenic *P. versicolora* larvae enhances mortality upon administration of lethal dsRNA

**Fig. 6:**
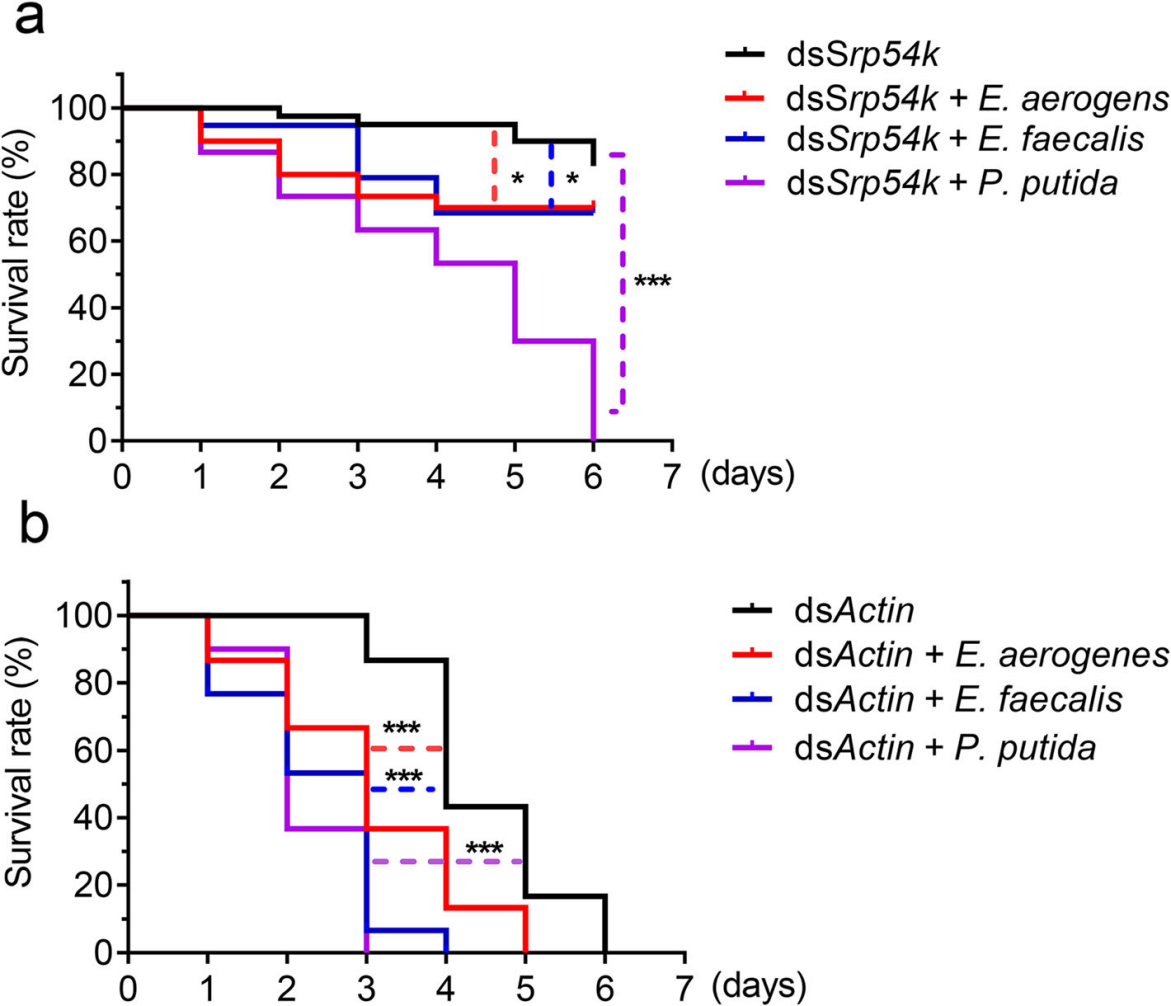
Kaplan–Meier survival curves of axenic *P. versicolora* larvae (*n* = 30) fed with ds*Srp54k* (**a**) or ds*Actin* (**b**) after reintroduction of bacterial species (*E. aerogenes*, *E. faecalis*, or *P. putida*). Shown here is one representative experiment of three repeats. The bacteria were mixed with either ds*Srp54k* or ds*Actin* and introduced into axenic larvae by spreading the bacteria onto aseptic poplar leaves. The log-rank test was used to assess the significance of differences between two survival curves. ^***^ *P* < 0.001, ^*^ *P* < 0.05

To ultimately confirm the impact of gut bacteria on dsRNA-induced killing, individual gut bacterial species (*P. putida, E. aerogenes* or *E. faecalis*) were mixed with lethal dsRNA and reintroduced into axenic larvae. Compared with axenic larvae, mortality was significantly increased in larvae inoculated with bacteria. Upon feeding of ds*Srp54k*, *P. putida* inoculated larvae were killed significantly faster than larvae inoculated with *E. aerogenes* or *E. faecalis* (Fig. 6a; log-rank test, *P* < 0.05). Similar results were obtained for ds*Actin* feeding. The accelerating effect of the gut bacteria was *P. putida* > *E. faecalis* > *E. aerogenes* (Fig. 6b; log-rank test, *P* < 0.05)*.*

## Discussion

In this work, we have explored whether the gut microbiota plays a role in dsRNA-mediated killing of pest insects by RNA interference (environmental RNAi). To this end, we developed a method to obtain axenic larvae. The method uses surface sterilization of eggs, which has several advantages compared with the antibiotic treatments that are often used in microbiome research to eliminate bacteria, including complete removal of microbes and absence of residual antibiotics that may affect larval development upon hatching. The axenic status can be maintained by feeding with aseptic leaves of poplar plants grown in a sterile envirionment. Feeding dsRNAs targeted against two essential genes (*β-Actin* and *Srp54k*) to axenic and non-axenic larvae of *P. versicolora*, we found that ds*Actin* is more effective in killing *P. versicolora* than ds*Srp54k* in either axenic or non-axenic conditions. The different killing efficiency might be due to the different cellular functions of the two proteins and/or differences in expression levels or transcript susceptibility to RNAi. Importantly, regardless of which dsRNA was fed, non-axenic larvae were killed significantly faster than axenic larvae. This effect was independent of the efficiency of gene knockdown (Fig. 1), excluding the possibility that the different mortalities are caused by different levels of gene silencing of the targeted genes.

To pinpoint potential causes of the accelerated mortality of non-axenic larvae, we determined abundance and composition of the gut microbiota in larvae after ingestion of dsRNA. Compared with control larvae, feeding of dsRNAs (irrespective of RNAi induction and target sequences, i.e., feeding with ds*GFP*, ds*Actin*, or ds*Srp54k*) resulted in significant alteration of the composition of the gut microbiota (Fig. 2) and excessive growth of gut bacteria (Fig. 3). The total bacterial load in ds*GFP* feeding larvae, however, decreased on day 5 to a similar level as in the control larvae (Fig. 3a, d).

The most abundant bacterial genus in the gut microbiota is *Enterobacter*, comprising over 80% of the total microbiota (Fig. 2c). In previous studies, *Enterobacter* was also found to be widely associated with leaf beetles and poplar pests [38, 39]. Bacteria of the genus *Rahnella* also could be readily isolated from *P. versicolora* using culture-dependent methods [19]. *Enterobacter* from the Colorado potato beetle was shown to suppress plant defense [39]; however, whether *Enterobacter* in *P. versicolora* has a similar function remains to be investigated. Notably, we found that there was a drastic increase in the abundance of *Pseudomonas* in larvae fed with ds*Srp54k* or ds*Actin* compared with larvae fed with ds*GFP* (Fig. 2c). Together with our observation that bacteria translocate to the hemocoel in *P. versicolora* larvae fed with lethal dsRNA, these findings indicate that the gut microbiota is an important contributor to dsRNA-mediated killing of pest insects.

**Fig. 7:**
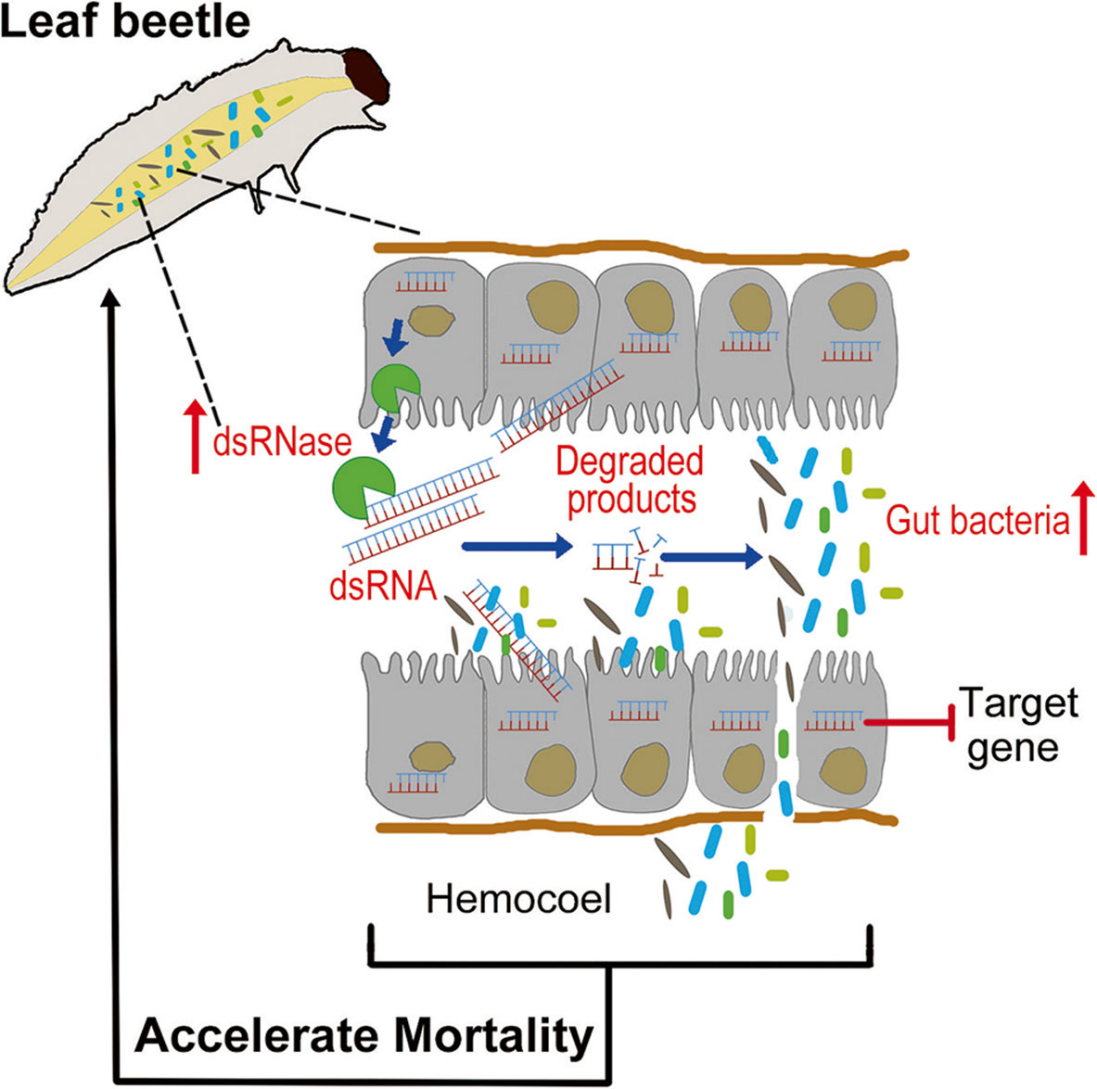
Working model of dsRNA-mediated insect killing. In the gut of the insect, dsRNA can be taken up by midgut epithelial cells and loaded onto the RNAi machinery, thus suppressing expression of the target gene. dsRNA can also induce expression of a gene encoding dsRNase, thereby causing degradation of dsRNA retained in the gut. Gut bacteria can utilize the degradation products of dsRNAs for their growth and translocate from the gut lumen into the hemocoel, thus resulting in accelerated mortality of their host insect

A previous study showed that dsRNA-mediated gene knockdown in the migratory locust can induce overgrowth of opportunistic pathogens and intestinal atrophy [40], but the reasons remained unclear. To clarify how RNAi influences gut bacteria, we analyzed bacterial growth in *P. versicolora* after ingestion versus injection of lethal dsRNA. We found that the knockdown of an essential gene has limited relevance to the increase in gut bacterial abundance (Fig. 4), in that *Srp54k* knockdown triggered by injection of ds*Srp54k* had no statistically significant effect on total bacterial growth. Similarly, the increase in bacterial abundance in larvae injected with ds*Actin* was lower than that in larvae fed with ds*Actin*. Thus, the direct comparison between ingestion and injection indicates that ingestion was required for accelerated mortality (Fig. 4). This finding raises the interesting question why oral administration leads to proliferation of the gut microbiota. As bacteria do not possess an RNAi machinery and no dsRNA uptake systems have been found in bacteria [41], we hypothesized that the dsRNA itself cannot trigger overgrowth of gut bacteria. A dsRNA-degradating enzyme (dsRNase) in the gut was first identified and characterized in *Bombyx mori*, and related enzymes were subsequently found in almost all orders of insects, including the beetle family (Coleoptera). Differences in the expression levels and activities of intestinal dsRNases are likely, at least in part, responsible for the observed differences in susceptibility to RNAi between different groups of insects [2, 7, 42–46]. In this work, we found that a gut dsRNase (dsRNase1) is chiefly responsible for dsRNA degradation in the digestive tract of *P. versicolora* (Fig. 5a–e). Moreover, we observed that ingestion of dsRNA upregulated *dsRNase1* expression (Fig. 5c). Suppressed expression of *dsRNase1* strongly reduced dsRNA degradation and decreased the abundance of gut bacteria (Fig. 5f, g), suggesting a contribution of *P. versicolora* dsRNase1 to growth control of gut bacteria. This finding also raised the interesting possibility that gut bacteria utilize the degradation products of dsRNAs as nutrient source (Fig. 5a). Evidence in support of this idea was supplied by bacterial growth assays that revealed that NTP mix, uridine, and inosine, but not dsRNA, can be utilized as carbon and nitrogen sources to promote the growth of three dominant bacterial strains in the gut microbiome of *P. versicolora* (Fig. 5h). Thus, ingested dsRNA can suffer one of two fates in the gut of *P. versicolora* larvae: (i) it can be taken up by midgut cells by a SID-type channel (putative dsRNA-selective transport channel) and/or the endocytosis pathway and subsequently trigger the RNAi response in the insect [47], or (ii) it undergoes degradation by dsRNase1 in the midgut (Fig. 5a) and its breakdown products are subsequently utilized by gut bacteria as carbon and nitrogen source for their growth (Figs. 5g and 7).

However, it seems reasonable to assume that dsRNA is not the only nutrient that controls gut bacterial growth and that there are likely other factors that contribute to the observed gut bacterial dysbiosis as the administration of ds*GFP* showed less bacterial growth than other two lethal dsRNA-fed larvae. For example, varied expression of gut antimicrobial peptides and other effectors, induced by oral feeding of dsRNA, could lead to differential survival of specific microbial taxa. Furthermore, since the increased mortality is seen only when specific (lethal) genes instead of ds*GFP* are ingested, it is likely that other mechanisms are at work besides the bacterial overgrowth. We found that the physiological structure of gut were disrupted in both lethal dsRNA-feeding larvae (Figure S4). However, whether or not immune activation or some other mechanisms lead to the disruption of gut structure needs further investigations. It is rational to conclude that the increased gut permeability and then translocation of specific gut bacteria into hemocoel partially contribute to the death of non-axenic *P. versicolora* when fed with lethal dsRNAs*.* Besides, it seems noteworthy that the capabililty of *P. putida* to utilize dsRNA degradation products as both carbon and nitrogen source for its growth is more pronounced than that of the other two abundant gut bacterial species tested (Fig. 5h; Table S3). This finding may, at least in part, explain why the proportion of *P. putida* was drastically increased in larvae fed with lethal dsRNAs (Fig. 2c). An alternative explanation is that *P. putida* may be more resistant than other gut bacteria to the immune response triggered by dsRNA ingestion. Moreover, reintroduction of gut bacteria into axenic larvae increased their mortality after feeding with lethal dsRNAs, and *P. putida* showed the strongest effect on acceleration of larval mortality (Fig. 6). *P. putida* was previously shown to be a nitrogen-fixing strain that can promote the growth of plants [48, 49], but whether the *P. putida* population in the gut microbiome of *P. versicolora* was originally acquired from its host plants and now functions as mildly pathogenic strain to *P. versicolora* remains to be investigated.

## Conclusions

In conclusion, the present study illuminates the complex interplay between lethal dsRNA, the insect host, and its gut microbiota (Fig. 7). We found that ingestion of dsRNA by insects caused a dysbiosis of gut bacterial community. The dsRNA degradation products by host insect preferentially promoted the growth of an entomopathogenic bacterium that contributed to the accelerated dsRNA lethality of the insect. Our work provides new insight into the interactions and suggests an important role of host sepsis in the multifaceted killing mechanism that underlies environmental RNAi.

## Supplementary Information

**Additional file 1: Figure S1.** Kaplan–Meier survival curves of *Plagiodera versicolora* larvae fed with poplar leaves that had been painted with identical amounts of the dsRNAs indicated (8 ng/cm^2^; black line: ds*GFP*; purple line: ds*Srp54k*; red line: ds*Actin*; blue line: ds*Shi*; green line: ds*Snap*, *n* = 30). The survival curves were analyzed by the log-rank test. **Figure S2.** Confirmation of the axenic status of *P. versicolora* larvae obtained from surface-sterilized eggs and influence of the gut microbiota on survival and growth of *P. versicolora* larvae. (A,B) The absence of bacteria from the axenically reared insects was confirmed by (A) the lack of bacterial colonies forming on LB agar plates (*n* = 10, gut extracts were diluted 10^4^ fold with sterilized H_2_O), and (B) the absence of PCR amplicons from reactions using universal 16S rRNA gene primers for bacteria (*n* = 10). PCR products were analyzed by gel electrophoresis in 1% agarose gels stained with ethidium bromide. M, DNA size marker; C, negative control (no template DNA added); L1 and L2, individual larva no. 1 and no. 2. (C) Kaplan–Meier survival curves of axenic and non-axenic *P. versicolora* larvae fed on aseptic poplar leaves for seven days (*n* = 30). The log-rank test was used to evaluate the significance of differences between the two groups. NS, not significant. (D) the weight of axenic and non-axenic *P. versicolora* larvae fed on aseptic poplar leaves for seven days. **Figure S3.** Translocation of gut bacteria to the hemocoel of *P. versicolora* larvae after ingestion of a lethal dose of dsRNA. Presence of bacteria was determined by plating homogenates of hemolymph fluid obtained from third-instar *P. versicolora* larvae fed with the indicated dsRNAs onto LB agar plates. Four representative plates are shown for each dsRNA treatment. Scale bars: 1 cm. **Figure S4.** Midgut morphology of non-axenic *P. versicolora* larvae fed with the indicated dsRNAs. Midgut cross-sections of larvae fed with ds*GFP* (A), ds*Srp54k* (B) or ds*Actin* (C) were stained with hematoxylin and eosin. Scale bars: 50 μm. **Figure S5.** Multiple sequence alignment of two *Plagiodera versicolora dsRNase* genes (Pv_dsRNase1 and Pv_dsRNase2) with the *dsRNase* genes from *Leptinotarsa decemlineata* (Ld_dsRNase1, KX652406; Ld_dsRNase2, KX652407), *Tribolium castaneum* (Tc_dsRNase1, XP_015840884; Tc_dsRNase2, XP_970494), *Locusta migratoria* (Lm_dsRNase, KX652408), *Schistocerca gregaria* (Sg_dsRNase1, AHN55088), and *Bombyx mori* (Bm_dsRNase1, AB254196). Identical and similar amino acid residues are shaded in black and gray, respectively. Conserved cysteine residues that may engage in disulfide bond formation are indicated by arrowheads. **Figure S6.** Expression profiles of two immune genes (*Duox1* and *Attacin 2*) in *Plagiodera versicolora* larvae after the ingestion of different dsRNAs for different times. (A) Gene expression level of *Duox1* and *Attacin2* after 2, 3, 4 and 5 days of feeding with ds*GFP*. (B) Gene expression level of *Duox1* and *Attacin2* upon ds*Srp54k* feeding. (C) Gene expression level of *Duox1* and *Attacin2* upon ds*Actin* feeding. Gene expression of each sample was normalized to that of the control treatment with ddH_2_O (set to 1). Four biological replicates were conducted. Data are presented as means ± SE, *P*-values were calculated using independent-samples t-test. *** *P* < 0.001; ** *P* < 0.01; * *P* < 0.05; NS, not significant. **Table S1.** Primers used in this study. **Table S2.** Comparison of diversity indices (mean ± SEM, *n* = 5) of the *P. versicolora* gut bacterial community in larvae fed with poplar leaves coated with H_2_O, ds*GFP*, ds*Srp54k* or ds*Actin*. **Table S3.** Abundances of bacterial genera in all samples. The abundance is presented as percentage of the total sequences in the sample. **Table S4.** Growth of three bacteria on RNA-related compounds. Each bacterium was grown for 24 h in liquid medium with a 24 nmol/mL concentration of each compound tested. ^1^(NH_4_)_2_SO_4_(0.4%)served as the nitrogen source. ^2^Glucose (0.4%) served as the carbon source.

## Data Availability

All data generated or analyzed during this study are included in this published article.
