# Supplementary material for: Synergistic action of the gut microbiota in environmental RNA interference in a leaf beetle
Source: Microbiome. 2021 May 4;9:98. doi: 10.1186/s40168-021-01066-1 (PMC8097945; doi:10.1186/s40168-021-01066-1)
Supplement: Supplementary file 2 — Additional file 1: Figure S1. Kaplan–Meier survival curves of Plagiodera versicolora larvae fed with poplar leaves that had been painted with identical amounts of the dsRNAs indicated (8 ng/cm2; black line: dsGFP; purple line: dsSrp54k; red line: dsActin; blue line: dsShi; green line: dsSnap, n = 30). The survival curves were analyzed by the log-rank test. Figure S2. Confirmation of the axenic status of P. versicolora larvae obtained from surface-sterilized eggs and influence of the gut microbiota on survival and growth of P. versicolora larvae. (A,B) The absence of bacteria from the axenically reared insects was confirmed by (A) the lack of bacterial colonies forming on LB agar plates (n = 10, gut extracts were diluted 104 fold with sterilized H2O), and (B) the absence of PCR amplicons from reactions using universal 16S rRNA gene primers for bacteria (n = 10). PCR products were analyzed by gel electrophoresis in 1% agarose gels stained with ethidium bromide. M, DNA size marker; C, negative control (no template DNA added); L1 and L2, individual larva no. 1 and no. 2. (C) Kaplan–Meier survival curves of axenic and non-axenic P. versicolora larvae fed on aseptic poplar leaves for seven days (n = 30). The log-rank test was used to evaluate the significance of differences between the two groups. NS, not significant. (D) the weight of axenic and non-axenic P. versicolora larvae fed on aseptic poplar leaves for seven days. Figure S3. Translocation of gut bacteria to the hemocoel of P. versicolora larvae after ingestion of a lethal dose of dsRNA. Presence of bacteria was determined by plating homogenates of hemolymph fluid obtained from third-instar P. versicolora larvae fed with the indicated dsRNAs onto LB agar plates. Four representative plates are shown for each dsRNA treatment. Scale bars: 1 cm. Figure S4. Midgut morphology of non-axenic P. versicolora larvae fed with the indicated dsRNAs. Midgut cross-sections of larvae fed with dsGFP (A), dsSrp54k (B) or dsActin (C) [file 40168_2021_1066_MOESM2_ESM.zip › Supporting information.docx]

**Supporting information**

**S1 Fig.** Kaplan–Meier survival curves of *Plagiodera versicolora* larvae fed with poplar leaves that had been painted with identical amounts of the dsRNAs indicated (8 ng/cm^2^; black line: ds*GFP*; purple line: ds*Srp54k*; red line: ds*Actin*; blue line: ds*Shi*; green line: ds*Snap*, n = 30). The survival curves were analyzed by the log-rank test.

**
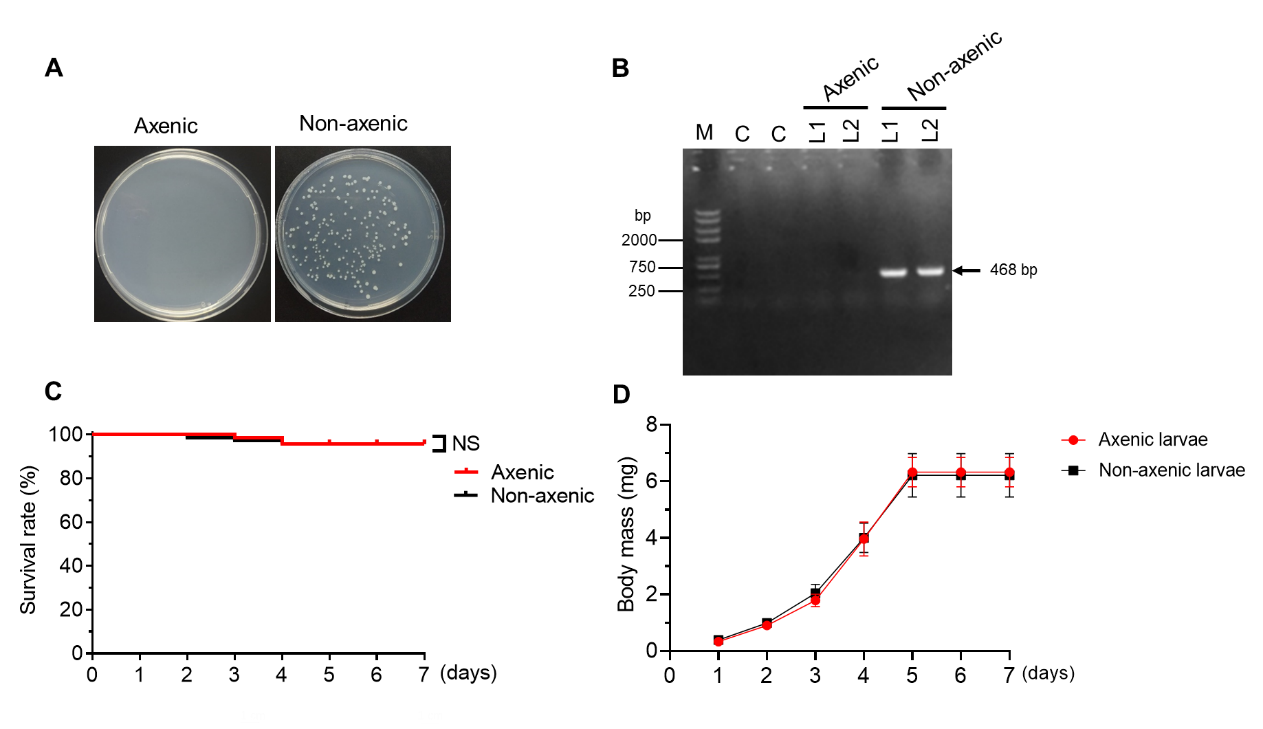
**

**S2 Fig.** Confirmation of the axenic status of *P. versicolora* larvae obtained from surface-sterilized eggs and influence of the gut microbiota on survival and growth of *P. versicolora* larvae. (A,B) The absence of bacteria from the axenically reared insects was confirmed by (A) the lack of bacterial colonies forming on LB agar plates (n = 10, gut extracts were diluted 10^4^ fold with sterilized H_2_O), and (B) the absence of PCR amplicons from reactions using universal 16S rRNA gene primers for bacteria (n = 10). PCR products were analyzed by gel electrophoresis in 1% agarose gels stained with ethidium bromide. M, DNA size marker; C, negative control (no template DNA added); L1 and L2, individual larva no. 1 and no. 2. (C) Kaplan–Meier survival curves of axenic and non-axenic *P. versicolora* larvae fed on aseptic poplar leaves for seven days (n = 30). The log-rank test was used to evaluate the significance of differences between the two groups. NS, not significant. (D) the weight of axenic and non-axenic *P. versicolora* larvae fed on aseptic poplar leaves for seven days.

**
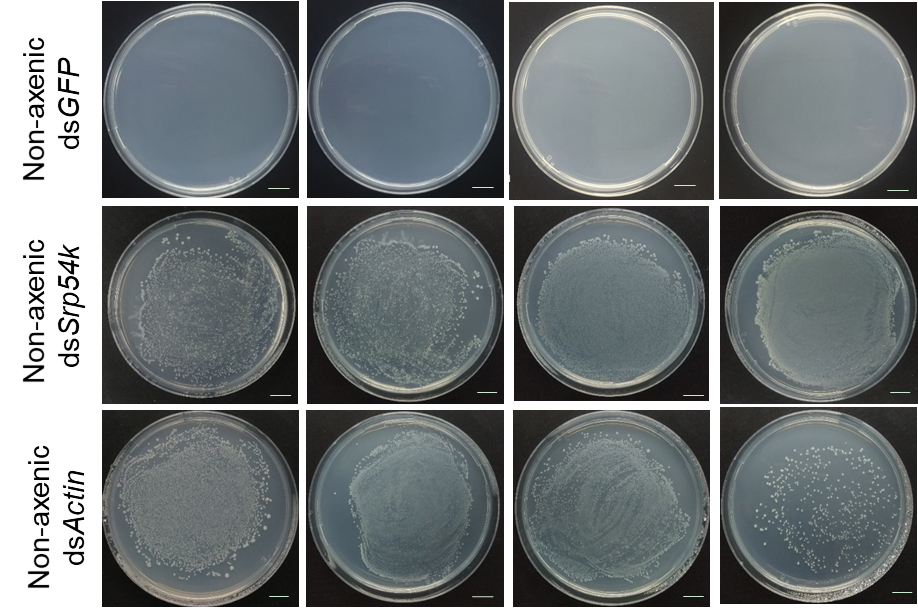
**

**S3 Fig**. Translocation of gut bacteria to the hemocoel of *P. versicolora* larvae after ingestion of a lethal dose of dsRNA. Presence of bacteria was determined by plating homogenates of hemolymph fluid obtained from third-instar *P. versicolora* larvae fed with the indicated dsRNAs onto LB agar plates. Four representative plates are shown for each dsRNA treatment. Scale bars: 1 cm.

**
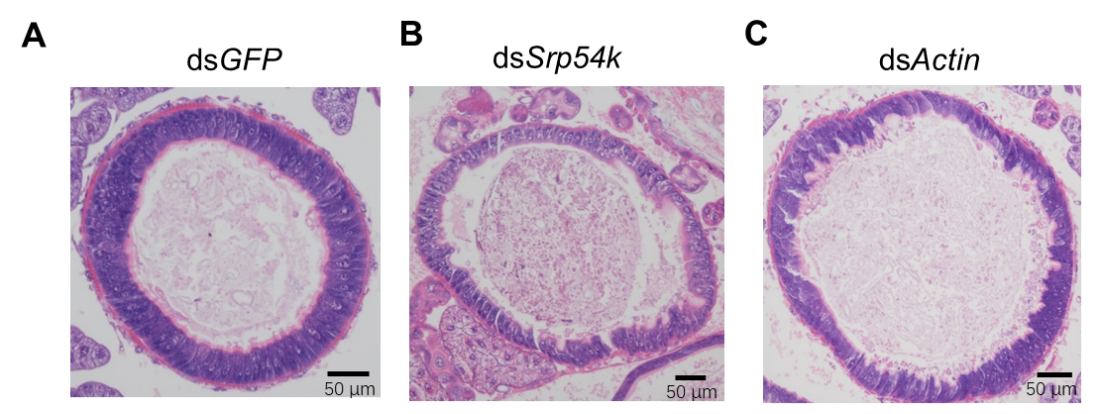
**

**S4 Fig.** Midgut morphology of non-axenic *P. versicolora* larvae fed with the indicated dsRNAs. Midgut cross-sections of larvae fed with ds*GFP* (A), ds*Srp54k* (B) or ds*Actin* (C) were stained with hematoxylin and eosin. Scale bars: 50 μm.


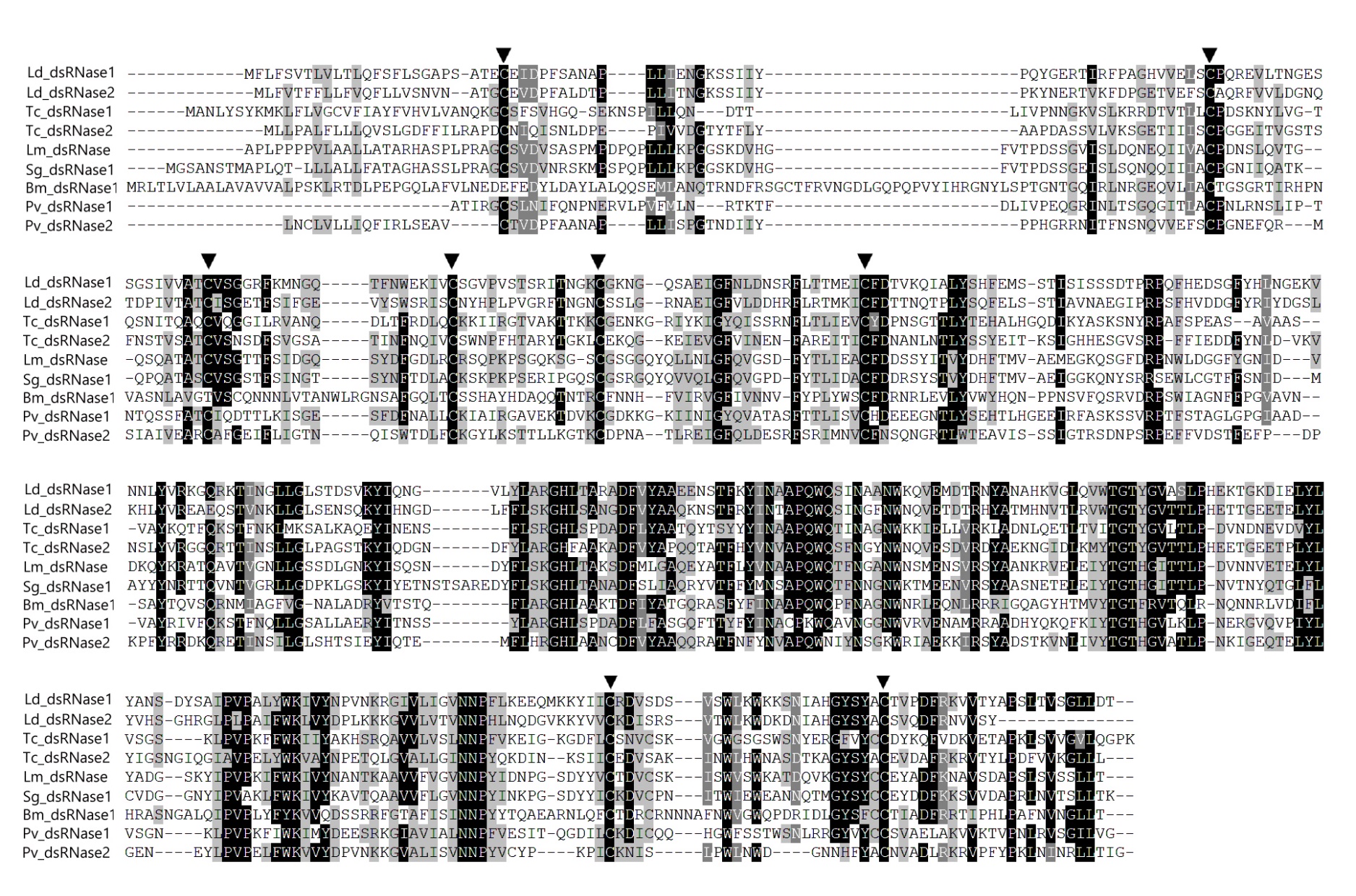


**S5 Fig.** Multiple sequence alignment of two *Plagiodera versicolora dsRNase* genes (Pv_dsRNase1 and Pv_dsRNase2) with the *dsRNase* genes from *Leptinotarsa decemlineata* (Ld_dsRNase1, KX652406; Ld_dsRNase2, KX652407), *Tribolium castaneum* (Tc_dsRNase1, XP_015840884; Tc_dsRNase2, XP_970494), *Locusta migratoria* (Lm_dsRNase, KX652408), *Schistocerca gregaria* (Sg_dsRNase1, AHN55088), and *Bombyx mori* (Bm_dsRNase1, AB254196). Identical and similar amino acid residues are shaded in black and gray, respectively. Conserved cysteine residues that may engage in disulﬁde bond formation are indicated by arrowheads.

**
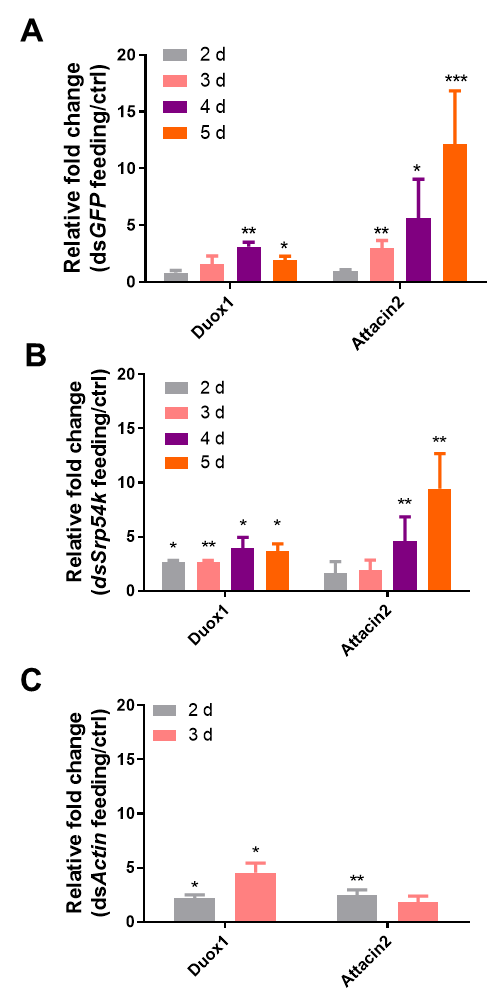
**

**S6 Fig.** Expression profiles of two immune genes (*Duox1* and *Attacin 2*) in *Plagiodera versicolora* larvae after the ingestion of different dsRNAs for different times. (A) Gene expression level of *Duox1* and *Attacin2* after 2, 3, 4 and 5 days of feeding with ds*GFP*. (B) Gene expression level of *Duox1* and *Attacin2* upon ds*Srp54k* feeding. (C) Gene expression level of *Duox1* and *Attacin2* upon ds*Actin* feeding. Gene expression of each sample was normalized to that of the control treatment with ddH_2_O (set to 1). Four biological replicates were conducted. Data are presented as means ± SE, *P*-values were calculated using independent-samples t-test. *** P < 0.001; ** P < 0.01; * P < 0.05; NS, not significant.

**S1Table.** Primers used in this study.

| **Primer** | **Sequence (5’-3’)** | **Application** |
| --- | --- | --- |
| 16S-27F | AGAGTTTGATCCTGGCTCAG | PCR of eubacterial 16S rRNA gene |
| 16S-1492R | TACGGYTACCTTGTTACGACTT |  |
| 16SF | TCCTACGGGAGGCAGCAGT | qPCR of eubacterial 16S rRNA gene |
| 16SR | GGACTACCAGGGTATCTAATCCTGTT |  |
| 338F | ACTCCTACGGGAGGCAGCA | PCR of V3 and V4 hypervariable regions of eubacterial 16S rRNA gene |
| 806R | GGACTACHVGGGTWTCTAAT |  |
| EnterococcusF | AGAAATTCCAAACGAACTTG | qPCR of *Enterococcus* genus |
| EnterococcusR | CAGTGCTCTACCTCCATCATT |  |
| PseudomonasF | ACTTTAAGTTGGGAGGAAGGG | qPCR of *Pseudomonas* genus |
| PseudomonaR | ACACAGGAAATTCCACCACCC |  |
| EnterobacterF | GTCTATTTCGCACGTCGTGCTTTG C | qPCR of *Enterobacter* genus |
| EnterobacterR | CTTCTCAACTGCGCGGATGAGACC |  |
| dsGFPF | GGATCCTAATACGACTCACTATAGGGAGATGGTGAGCAAGGGCGAGGAGCTGT | RNAi of GFP as negative control |
| dsGFPR | GGATCCTAATACGACTCACTATAGGGAGTTACTTGTACAGCTCGTCCATGCCG |  |
| dsSrp54kF | GGATCCTAATACGACTCACTATAGGGCGGCTGACACAATGGATAAACA | RNAi of beetle *Srp54k* |
| dsSrp54kR | GGATCCTAATACGACTCACTATAGGTGGATCCTGTGGTTATCGCT |  |
| dsActinF | GGATCCTAATACGACTCACTATAGGACCCCTGCCATGTACGTCGCTA | RNAi of beetle *Actin* |
| dsActinR | GGATCCTAATACGACTCACTATAGGCCGATGGTGATGACTTGTCCG |  |
| dsShiF | GGATCCTAATACGACTCACTATAGGCCCGCAGCTCAAGAGGCAGGT | RNAi of beetle *Shi* |
| dsShiR | GGATCCTAATACGACTCACTATAGGACCGCTGTAGATTTTCCACCAC |  |
| dsSnapF | GGATCCTAATACGACTCACTATAGGTTGAGAAGGCATTTGTTAGCTGAA | RNAi of beetle *Snap* |
| dsSnapR | GGATCCTAATACGACTCACTATAGGAATGTTACCAAAGAGCAGCAAATC |  |
| dsdsRNase1F | GGATCCTAATACGACTCACTATAGGACGCTACATCACCAACAG | RNAi of beetle *dsRNase1* |
| dsdsRNase1R | GGATCCTAATACGACTCACTATAGGCCTTCCTGGACTCTTCATC |  |
| dsdsRNase2F | GGATCCTAATACGACTCACTATAGGCAGTGGAAGTGTTACTCAAG | RNAi of beetle *dsRNase2* |
| dsdsRNase2R | GGATCCTAATACGACTCACTATAGGTATTGTGGAAGCAAGATGTG |  |
| Srp54kF | ACTCCTCCTGCTTATGTCTACC | qPCR of *Srp54k* |
| Srp54kR | TCCTGTGGTTATCGCTCAAGA |  |
| ActinF | CGTGACTTGACCGACTACCT | qPCR of *Actin* |
| ActinR | CGAGAGCGACATAGCAGAGT |  |
| ShiF | TCAAGAGGCAGGTGGAGAC | qPCR of *Shi* |
| ShiR | TCTTCGGCACCAAGTCCTT |  |
| SnapF | AACAGTTCACTGCCTCATTGAT | qPCR of *Snap* |
| SnapR | TGGTGCCAGACATGATGCT |  |
| dsRNase1F | TCACGACGAGGAGGAAGGA | qPCR of *dsRNase1* |
| dsRNase1R | GGACGAACTGAGGTCTTGGA |  |
| dsRNase2F | CTATACGCCACTTGCCTGAAT | qPCR of *dsRNase2* |
| dsRNase2R | GGTCACCTAGCAGCCAATG |  |
| Duox1 | GCAACTACAACGCAGAAGATCA | qPCR of *Duox* |
| Duox1 | GACACCGATACCACCTCCTAC |  |
| Attacin | GTCCTTTGCCACCGTTAGC | qPCR of *Attacin* |
| Attacin | TGGGAAATTGGGTTTGTCTCAT |  |

**S2 Table.** Comparison of diversity indices (mean ± SEM, n = 5) of the *P. versicolora* gut bacterial community in larvae fed with poplar leaves coated with H_2_O, ds*GFP*, ds*Srp54k* or ds*Actin*.

| **Index** | **H_2_O** | **ds*GFP*** | **ds*Srp54k*** | **ds*Actin*** |
| --- | --- | --- | --- | --- |
| ACE diversity | 41.45±14.67 | 30.31±2.15 | 29.80±5.44 | 43.27±3.80 |
| Chao1 diversity | 39.78±14.65 | 29.22±3.34 | 31.19±5.73 | 38.10±2.56 |
| Shannon diversity (H) | 0.50±0.12^a^ | 0.78±0.07^a^ | 0.78±0.14^a^ | 1.34±0.06^b^ |
| Simpson’s diversity | 0.80±0.04^a^ | 0.61±0.04^a^ | 0.60±0.09^a^ | 0.35±0.02^b^ |

OTU richness and diversity values were calculated for genus-level OTUs.

Data were analyzed using one-way ANOVA followed by Bonferroni’s multiple comparisons. Superscript letters denote significant differences at *P* ≤ 0.05.

**S3 Table.** Abundances of bacterial genera in all samples. The abundance is presented as percentage of the total sequences in the sample.

**S4 Table.** Growth of three bacteria on RNA-related compounds. Each bacterium was grown for 24 h in liquid medium with a 24 nmol/mL concentration of each compound tested.

^1^(NH_4_)_2_SO_4_（0.4%）served as the nitrogen source.

^2^Glucose (0.4%) served as the carbon source.

| **Bacterial cells/mL (10^-8^) (mean** ± **SEM)** | | | | | | |
| --- | --- | --- | --- | --- | --- | --- |
| **Growth substrate** | **Carbon source^1^** | | | **Nitrogen source^2^** | | |
| ID | *Pseudomonas putide* | *Enterobacter aerogenes* | *Enterococcus faecalis* | *Pseudomonas putide* | *Enterobacter*  *aerogenes* | *Enterococcus faecalis* |
| None | 0.96±0.17 | 7.36±0.59 | 0.73±0.12 | 12.73±0.90 | 4.91±0.13 | 3.03±0.46 |
| dsGFP | 0.97±0.12 | 7.41±2.95 | 0.76±0.15 | 11.80±0.44 | 4.92±0.22 | 3.03±0.24 |
| Ribose | 6.83±0.94 | 11.49±0.58 | 0.70±0.12 | 11.67±2.34 | 4.20±0.64 | 3.67±0.09 |
| Adenine | 1.03±0.18 | 9.87±0.83 | 0.63±0.10 | 86.67±6.92 | 7.30±0.49 | 73.93±3.54 |
| Adenosine | 8.33±0.24 | 11.72±0.67 | 1.50±0.12 | 54.93±6.76 | 10.47±1.03 | 39.27±6.21 |
| Cytidine | 8.40±0.81 | 10.67±2.20 | 2.13±0.34 | 40.40±4.76 | 8.89±1.22 | 20.45±2.76 |
| Uridine | 7.37±0.75 | 9.69±1.16 | 0.80±0.15 | 27.07±4.39 | 6.23±0.65 | 21.10±1.95 |
| Inosine | 13.73±2.58 | 9.51±1.53 | 0.60±0.10 | 38.83±1.39 | 7.30±0.34 | 34.17±4.65 |
